# Exposure to PM_2.5_ is a risk factor for acute exacerbation of surgically diagnosed idiopathic pulmonary fibrosis: a case–control study

**DOI:** 10.1186/s12931-021-01671-6

**Published:** 2021-03-12

**Authors:** Masahiro Tahara, Yoshihisa Fujino, Kei Yamasaki, Keishi Oda, Takashi Kido, Noriho Sakamoto, Toshinori Kawanami, Kensuke Kataoka, Ryoko Egashira, Mikiko Hashisako, Yuzo Suzuki, Tomoyuki Fujisawa, Hiroshi Mukae, Takafumi Suda, Kazuhiro Yatera

**Affiliations:** 1 Department of Respiratory Medicine, University of Occupational and Environmental Health, Japan, 1-1 Iseigaoka, Yahatanishi-ku, Kitakyushu-city, Fukuoka 807-8555 Japan; 2 Department of Environmental Epidemiology, Institute of Industrial Ecological Sciences, University of Occupational and Environmental Health, Japan, Kitakyushu, Japan; 3 Department of Respiratory Medicine, Nagasaki University Graduate School of Biomedical Sciences, Nagasaki, Japan; 4 Department of Respiratory Medicine and Allergy, Tosei General Hospital, Seto, Japan; 5 Department of Radiology, Faculty of Medicine, Saga University, Saga, Japan; 6 Department of Anatomic Pathology, Graduate School of Medical Sciences, Kyushu University, Fukuoka, Japan; 7 Second Division, Department of Internal Medicine, Hamamatsu University School of Medicine, Hamamatsu, Japan

**Keywords:** Acute exacerbation, Air pollution exposure, Idiopathic pulmonary fibrosis, Particulate matter, Risk factors

## Abstract

**Background:**

Short-term exposure to ozone and nitrogen dioxide is a risk factor for acute exacerbation (AE) of idiopathic pulmonary fibrosis (AE-IPF). The comprehensive roles of exposure to fine particulate matter in AE-IPF remain unclear. We aim to investigate the association of short-term exposure to fine particulate matter with the incidence of AE-IPF and to determine the exposure-risk time window during 3 months before the diagnosis of AE-IPF.

**Methods:**

IPF patients were retrospectively identified from the nationwide registry in Japan. We conducted a case–control study to assess the correlation between AE-IPF incidence and short-term exposure to eight air pollutants, including particulate matter < 2.5 µm (PM_2.5_). In the time-series data, we compared monthly mean exposure concentrations between months with AE (case months) and those without AE (control months). We used multilevel mixed-effects logistic regression models to consider individual and institutional-level variables, and also adjusted these models for several covariates, including temperature and humidity. An additional analysis with different monthly lag periods was conducted to determine the risk-exposure time window for 3 months before the diagnosis of AE-IPF.

**Results:**

Overall, 152 patients with surgically diagnosed IPF were analyzed. AE-IPF was significantly associated with an increased mean exposure level of nitric oxide (NO) and PM_2.5_ 30 days prior to AE diagnosis. Adjusted odds ratio (OR) with a 10 unit increase in NO was 1.46 [95% confidence interval (CI) 1.11–1.93], and PM_2.5_ was 2.56 (95% CI 1.27–5.15). Additional analysis revealed that AE-IPF was associated with exposure to NO during the lag periods lag 1, lag 2, lag 1–2, and lag 1–3, and PM_2.5_ during the lag periods lag 1 and lag 1–2.

**Conclusions:**

Our results show that PM_2.5_ is a risk factor for AE-IPF, and the risk-exposure time window related to AE-IPF may lie within 1–2 months before the AE diagnosis. Further investigation is needed on the novel findings regarding the exposure to NO and AE-IPF.

**Supplementary Information:**

The online version contains supplementary material available at 10.1186/s12931-021-01671-6.

## Background

Idiopathic pulmonary fibrosis (IPF) is a fibrotic lung disease characterized by the progressive impairment of lung function and poor prognosis [1]. The natural history of IPF is heterogeneous and its median survival is 2–5 years [1–3]. The associations of increased exposure levels of particulate matter < 2.5 µm (PM_2.5_) and particulate matter < 10 µm (PM_10_) and the mortality of patients with IPF are reported, but not correlated with the incidence of acute exacerbation (AE) of IPF (AE-IPF) [4]. The two antifibrotic drugs pirfenidone and nintedanib have recently been identified for the treatment of IPF [5, 6]. Several registries have shown the survival benefits of these two antifibrotic drugs on patients with IPF [7, 8]; however, IPF still remains a life-threatening disease.

Some patients with IPF experience severe deteriorations that are associated with rapid disease progression and high mortality, which is termed AE-IPF [9, 10]. AE-IPF is defined as the acute worsening of respiratory symptoms combined with new radiographic lung opacities on high-resolution computed tomography (HRCT) without any identifiable causes [9, 10]. The most common cause of death in Japanese patients with IPF is AE [3]. In addition, the percentage of AE-related deaths (40%) in Japan is reportedly higher than that observed in Western countries (18%) [3]. Viral infection [11], microaspiration [12] and SLB [13] are established triggers for AE-IPF. Regarding exposure to ambient air pollution as potential risk factors of AE-IPF, a significant association between the incidence of AE-IPF and increased mean exposure levels of ozone (O_3_) and nitrogen dioxide (NO_2_) during 0–42 days prior to AE was shown in South Korea [14]. Similarly, a French study showed that short-term exposure to increased level of O_3_ was positively related to AE-IPF [4].

Among ambient air pollutants, fine particulate matter is considered as particularly dangerous. Indeed, an association has been shown between the elevated levels of airborne fine particulate matter and the risk of hospital admissions for patients with asthma, chronic obstructive pulmonary disease (COPD) and cardiovascular disease [15–18]. Dales et al. demonstrated that exposure to fine particulate matter is a risk factor for hospitalization of patients with IPF [19]. The two previous studies (South Korea and France) suggested that fine particulate matter may be a risk factor for AE-IPF; however, their results were not statistically significant [4, 14]. Moreover, these studies applied the exposure time window as “the 0–42 days” prior to the diagnosis of AE according to the definition of AE-IPF (i.e. onset should be within 1 month prior to diagnosing AE-IPF) [4, 9, 10, 14]. However, the specific exposure time window during which patients with IPF are at risk of developing AEs remains undefined.

We hypothesized that the short-term exposure to fine particulate matter could increase the incidence of AE-IPF. Therefore, we investigated whether the incidence of AE-IPF associated with increased mean exposure level of fine particulate matter in the month with AE. Furthermore, we performed an additional analysis with different monthly lag periods to determine the exposure-risk time window during 3 months before the diagnosis of AE. We also investigated the correlations between the AE of idiopathic interstitial pneumonias (AE-IIPs) and the air pollutant exposure levels. The exposure levels of eight air pollutants, namely sulfur dioxide (SO_2_), nitric oxide (NO), NO_2_, nitrogen oxides (NO_X_), carbon monoxide (CO), O_3_, PM_2.5_, and PM_10_, were evaluated using the nationwide surgically diagnosed IPF and IIPs registry in Japan [20].

## Methods

### Study design

We performed a case–control study to investigate the correlation between short-term exposure to air pollutants and the incidence of AE. A time-series data was used to compare monthly mean exposure concentrations between 41 months with AE (case months) and 5742 months without AE (control months) (Additional file 1: Fig. S1). Using a multilevel approach, we adjusted the effects for individual confounders because this case–control study included the individual time-series data such as air pollutant levels. Finally, we adjusted the effects for individual and institution confounders using the multilevel mixed-effects logistic regression models with a two-level structure of patients nested within the 33 hospitals [21, 22]. Although case–crossover is the most common design for analyzing the health-related effects of air pollution [23], this statistical methodology (the case–control study partially including case–crossover design) allowed us to use all the single months without AE as controls (Additional file 1: Fig. S1).

### Source database and study subjects

Japanese patients were identified from the nationwide cloud-based integrated database for IIPs in Japan [20]. In the online database, 465 patients with an institutional diagnosis of IIPs who had undergone chest HRCT and SLB from April 2009 to March 2014 were retrospectively collected from 39 institutions. Subsequently, a cloud-based MDD involving respiratory physicians, radiologists and pathologists with expertise in interstitial lung disease (ILD) was conducted via video-conferencing according to the International IPF statements and IIPs classification (see Additional file 1: Methods) [1, 24, 25]. From the database, we excluded patients who met the following criteria: (a) MDD diagnosis was not an IIPs; (b) the corresponding ambient air pollution data were unavailable (patients registered before 2008 were excluded due to unavailable air pollution data from the nationwide database); (c) AE occurred within 2 months after the SLB procedure and (d) follow-up period was < 2 months.

AE-IPF was diagnosed based on the following criteria established by the American Thoracic Society/European Respiratory Society [9]: (1) within 1 month of the clinical course of IPF disease progression, the following two conditions should have been satisfied: (a) worsening of dyspnea and (b) presence of new ground-glass opacities on chest HRCT and (2) exclusion of other identifiable causes [9]. Patients with AE-IIPs were diagnosed based on the criteria for AE-IPF [9]. Among patients who experienced ≥ 2 AEs, only the first event was included in the analysis. This study was conducted in accordance with the amended Declaration of Helsinki. The institutional review boards of the University of Occupational and Environmental Health, Kitakyushu, Japan (18-013) and the Hamamatsu University School of Medicine, Hamamatsu, Japan (19-003) approved the protocol.

### Measurement

We extracted the following information at the time of SLB from the database as baseline characteristics: age, sex, smoking status (pack-years), percentage of predicted value of forced vital capacity (FVC), percentage of predicted value of diffusing capacity of the lung for carbon monoxide (DL_CO_), HRCT pattern [1], and data pertaining to therapy with antifibrotic drugs (e.g., pirfenidone and nintedanib). The date of the SLB procedure and the diagnosis of AE from April 1, 2009, to March 31, 2017, were obtained from the database.

Daily and monthly mean concentrations of SO_2_, NO, NO_2_, NO_X_, CO, O_3_, PM_2.5_ and PM_10_ were obtained from the nationwide database of the Atmospheric Environment Regional Observation System using the website of the National Institute for Environmental Studies, Japan (http://www.nies.go.jp/igreen/index.html). O_3_ measurements were obtained during the 15-h period of daylight (5:00–20:00). For the PM_10_ measurements, the measurements obtained as suspended particulate matter (SPM) from the website were used because SPM is defined as airborne particulate matter with a diameter smaller than or equal to 10 in Japan. Daily and monthly mean temperature and humidity values were obtained from the database of the Japan Meteorological Agency (https://www.data.jma.go.jp/gmd/risk/obsdl/index.php). We selected the air monitoring stations located nearest to the registered hospitals from 1907 air monitoring stations in Japan and obtained the levels of air pollutants, temperature, and humidity. The patients who developed AE were admitted to these registered hospitals. Demographic data for each prefecture and neighborhood-level factors in Japan were obtained from the 2015 national census (https://www.e-stat.go.jp/).

### Statistical analysis

Multilevel mixed-effects logistic regression models were used to evaluate the association between the incidence of AE-IPF and monthly mean exposure for each air pollutant by matching data on the case month with that on the control month. For each AE diagnosis, the case month was defined as 30 days before AE diagnosis (Additional file 1: Fig. S1). The control months were defined as all the single months during the date of SLB procedure to the date of death or censoring in patients without AE. In patients with AE, we served all the single months other than the case month as control months (see Additional file 1: Methods and Fig. S1). Patients who were alive without the incidence of AE on March 31, 2017 were censored. Patients lost to follow-up were censored at the date of last contact/follow-up. We adjusted the effects for individual and institution confounders using the multilevel regression models with a two-level structure of patients nested within the 33 hospitals [21, 22].

In addition, we estimated the single month lag exposure (lag 1 to lag 3) and cumulative exposure (lag 1–2 to lag 1–3). For example, lag 1 exposure refers to the exposure during 30 days prior to AE diagnosis, while lag 1–2 exposure refers to the exposure during 60 days before AE diagnosis. The definition of control and case periods in the additional analysis is shown in Additional file 1: Fig. S2.

These models were adjusted for temperature, humidity, age, sex, smoking status (pack-years), the percentage of the predicted value of FVC and DL_CO_ at the time of SLB (risk factors identified in previous studies [26]), and neighborhood-level factors, such as regional characteristics of population density and per capita income [27]. Results are presented as odds ratios (ORs) with 95% confidence intervals (CIs) associated with a 10-unit increase in SO_2_ (ppb), NO (ppb), NO_2_ (ppb), NO_X_ (ppb), CO (ppb), O_3_ (ppb), PM_2.5_ (µg/m^3^) and PM_10_ (µg/m^3^). We also assessed the association between the incidence of AE-IIPs and monthly mean exposure for each air pollutant using the same methodology used for AE-IPF. All analyses were conducted at a significant α level of 0.05. All statistical analyses were performed using the STATA 16.1 software (StataCorp, College Station, TX, USA). Complete details of the methods are available in the Additional file 1: Material.

## Results

**Fig. 1:**
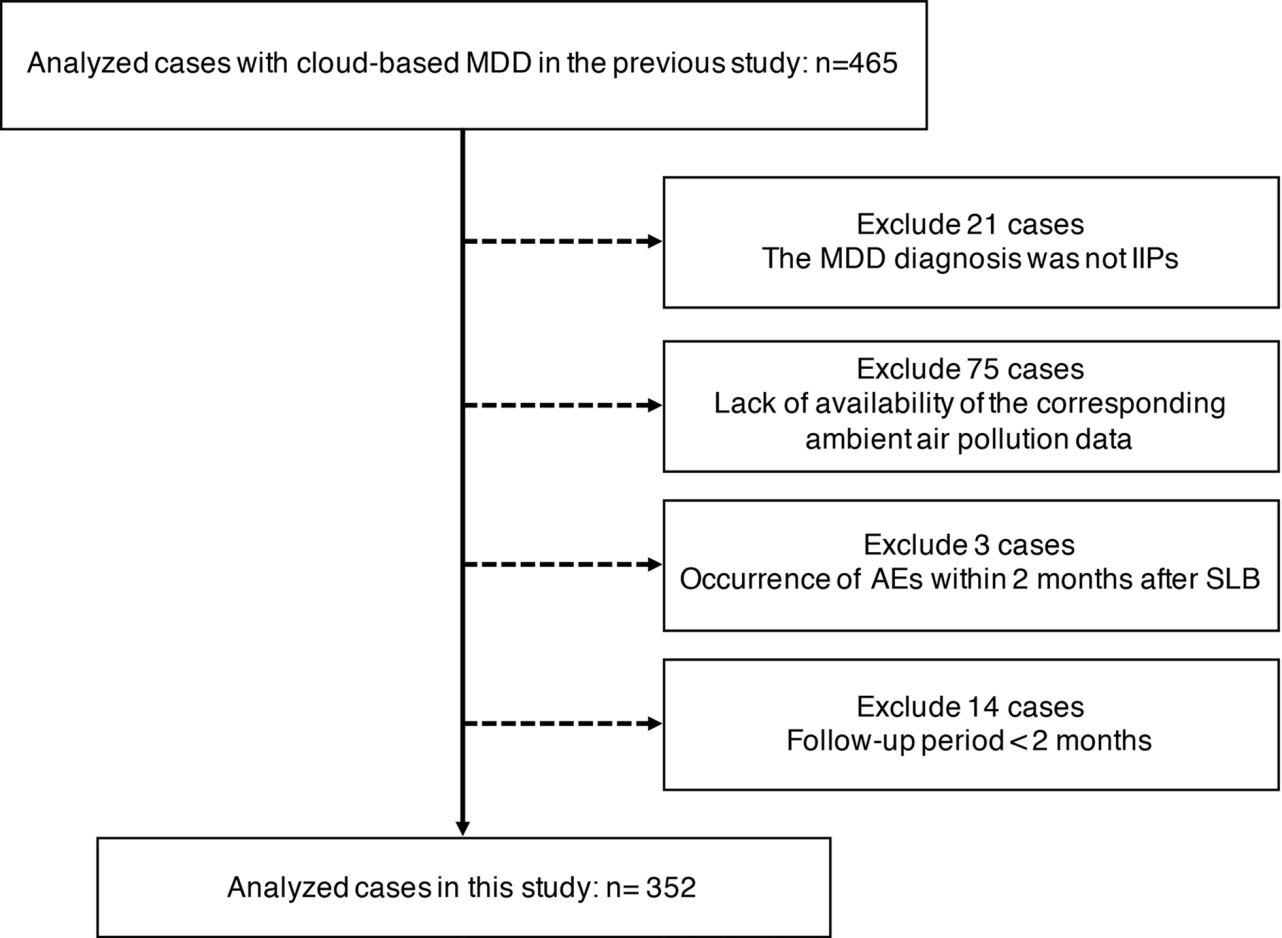
Patient flow diagram. *MDD* multidisciplinary discussion, *IIPs* idiopathic interstitial pneumonias, *AE* acute exacerbation, *SLB* surgical lung biopsy

**Fig. 2:**
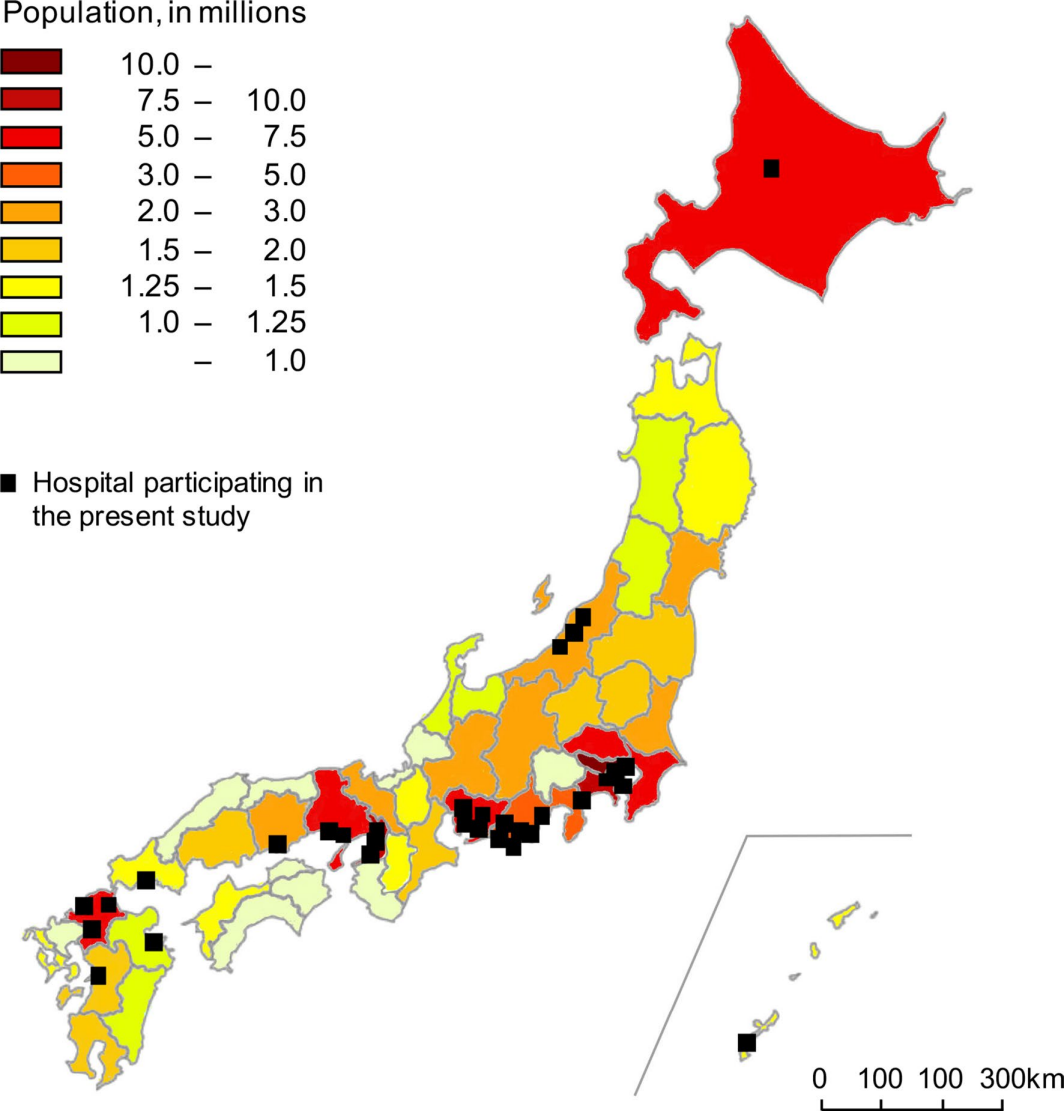
Demographic data and geographic location of the 33 hospitals. The demographic data in 2015 in Japan is shown in different colors for each prefecture. The numbers on the top left represent the population and correspond to each color. The black boxes indicate the geographic location of 33 Japanese hospitals participating in the present study

Among the 465 patients included in the previous study [20], 113 were excluded for the following reasons: MDD diagnosis was not an IIPs (n = 21); lack of the corresponding ambient air pollution data (n = 75); AE occurred within 2 months after SLB (n = 3); follow-up period was < 2 months (n = 14) (Fig. 1). Finally, 352 patients with IIPs (152 IPF, 35 idiopathic nonspecific interstitial pneumonia, four cryptogenic organizing pneumonia, seven desquamative interstitial pneumonia/respiratory bronchiolitis-ILD and 15 idiopathic pleuroparenchymal fibroelastosis and 139 unclassifiable IIPs) were enrolled in the present study (Table 1). Baseline (at SLB) clinical characteristics of the participants with or without AE-IPF and AE-IIPs are shown in Table 2 and Additional file 1: Table S1, respectively. The proportion of the UIP pattern in HRCT was 15% of the IPF patients (Table 2). Figure 2 shows the demographic data and the locations of the 33 hospitals participating in the present study; all monitoring stations were located within 10-km radius of the hospitals (Additional file 1: Figs. S3 and S4–6). The distribution of meteorological and air pollutant-exposure levels in patients with IPF and IIPs are shown in Table 3 and Additional file 1: Table S2, respectively.

**Table 1:** Number of patients diagnosed through MDD in the present study

MDD diagnosis	All	Acute exacerbation	No acute exacerbation
IIPs	352	74	278
IPF	152	41	111
iNSIP	35	6	29
COP	4	0	4
DIP/RB-ILD	7	1	6
iPPFE	15	5	10
Unclassifiable IIPs	139	21	118

Data presented as frequencies. *MDD* multidisciplinary discussion, *IIPs* idiopathic interstitial pneumonias, *IPF* idiopathic pulmonary fibrosis, *iNSIP* idiopathic nonspecific interstitial pneumonia, *COP* cryptogenic organizing pneumonia, *DIP/RB-ILD* desquamative interstitial pneumonia/respiratory bronchiolitis-interstitial lung disease, *iPPFE* idiopathic pleuroparenchymal fibroelastosis

**Table 2:** Characteristics of patients with IPF at the time of SLB

	All	Acute exacerbation	No acute exacerbation
Subjects	152	41	111
Case or control months	5,783	41	5,742
Age (years)	65 [61–70]	68 [63–71]	65 [60–70]
Male	106 (70%)	29 (71%)	77 (69%)
Pack-years	30 [0–45]	30 [0–53]	26 [0–45]
Pulmonary function tests			
FVC (% predicted)	83.9 [75.1–96.4]	83.6 [75–92.2]	85.2 [75.2–97.2]
DL_CO_ (% predicted)	71.6 [55.5–85.6]	61.0 [48.5–75.9]	74.8 [57.2–88.4]
HRCT pattern			
UIP	23 (15%)	9 (22%)	14 (13%)
Possible UIP	122 (80%)	30 (73%)	92 (83%)
Inconsistent with UIP	7 (5%)	2 (5%)	5 (4%)
Antifibrotic therapy			
Pirfenidone	55 (36%)	18 (44%)	37 (33%)
Nintedanib	19 (13%)	3 (7%)	16 (14%)

Data presented as median [interquartile rage] or frequencies (%)

*IPF* idiopathic pulmonary fibrosis, *SLB* surgical lung biopsy, *FVC* forced vital capacity, *DL*_*CO*_ diffusing capacity of the lung for carbon monoxide, *HRCT* high-resolution computed tomography

**Table 3:** Distribution of meteorological and air pollutant exposure levels in patients with IPF

Air pollutants	Acute exacerbation				No acute exacerbation			
	Mean	Min	Median	Max	Mean	Min	Median	Max
Temperature (°C)	14.1	− 3.3	13.4	28.0	16.3	− 9.4	16.4	30.0
Humidity (%)	67.4	42.7	68.6	87.0	66.8	36.0	67.0	87.0
SO_2_ (ppb)	2.6	0.0	2.6	12.5	2.8	0.0	3.0	8.0
NO (ppb)	10.8	1.1	3.4	98.0	5.6	1.0	4.0	69.0
NO_2_ (ppb)	16.7	3.1	14.7	37.0	14.9	2.0	14.0	46.0
NO_X_ (ppb)	26.4	2.5	16.5	133.0	20.5	4.0	17.0	106.0
CO (ppb)	460.4	81.6	412.1	2101.0	411.4	100.0	400.0	1700.0
O_3_ (ppb)	28.0	6.2	28.2	47.6	29.2	5.0	28.0	57.0
PM_2.5_ (µg/m^3^)	17.0	6.0	16.8	42.6	15.3	1.6	14.8	43.6
PM_10_ (µg/m^3^)	20.1	7.7	19.3	30.3	20.7	6.0	20.0	51.0

*SO*_2_ sulfur dioxide, *NO* nitric oxide, *NO*_2_ nitrogen dioxide, *NO*_*X*_ nitrogen oxides, *CO* carbon monoxide, *O*_3_ ozone, *PM*_2.5_ particulate matter < 2.5 µm, *PM*_10_ particulate matter < 10 µm

**Fig. 3:**
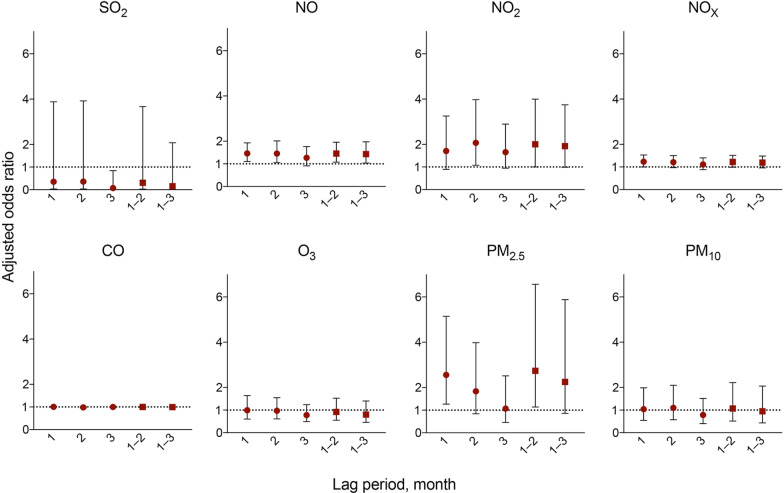
Adjusted ORs and 95% CIs for the incidence of AE-IPF with different monthly lag periods. Adjusted odds ratios (ORs) and 95% confidence intervals (CIs) showing the increased risk of acute exacerbation of idiopathic pulmonary fibrosis (AE-IPF) associated with a 10-unit increase in the levels of sulfur dioxide (SO_2_), nitric oxide (NO), nitrogen dioxide (NO_2_), nitrogen oxides (NO_X_), carbon monoxide (CO), ozone (O_3_), particulate matter < 2.5 µm (PM_2.5_) and particulate matter < 10 µm (PM_10_) with different monthly lag periods. The adjusted ORs were estimated using multilevel mixed-effects logistic regression models, adjusting for temperature, humidity, age, sex, smoking status (pack-years) and percentage of the predicted value of forced vital capacity and diffusing capacity of the lung for carbon monoxide, and neighbourhood-level factors

**Fig. 4:**
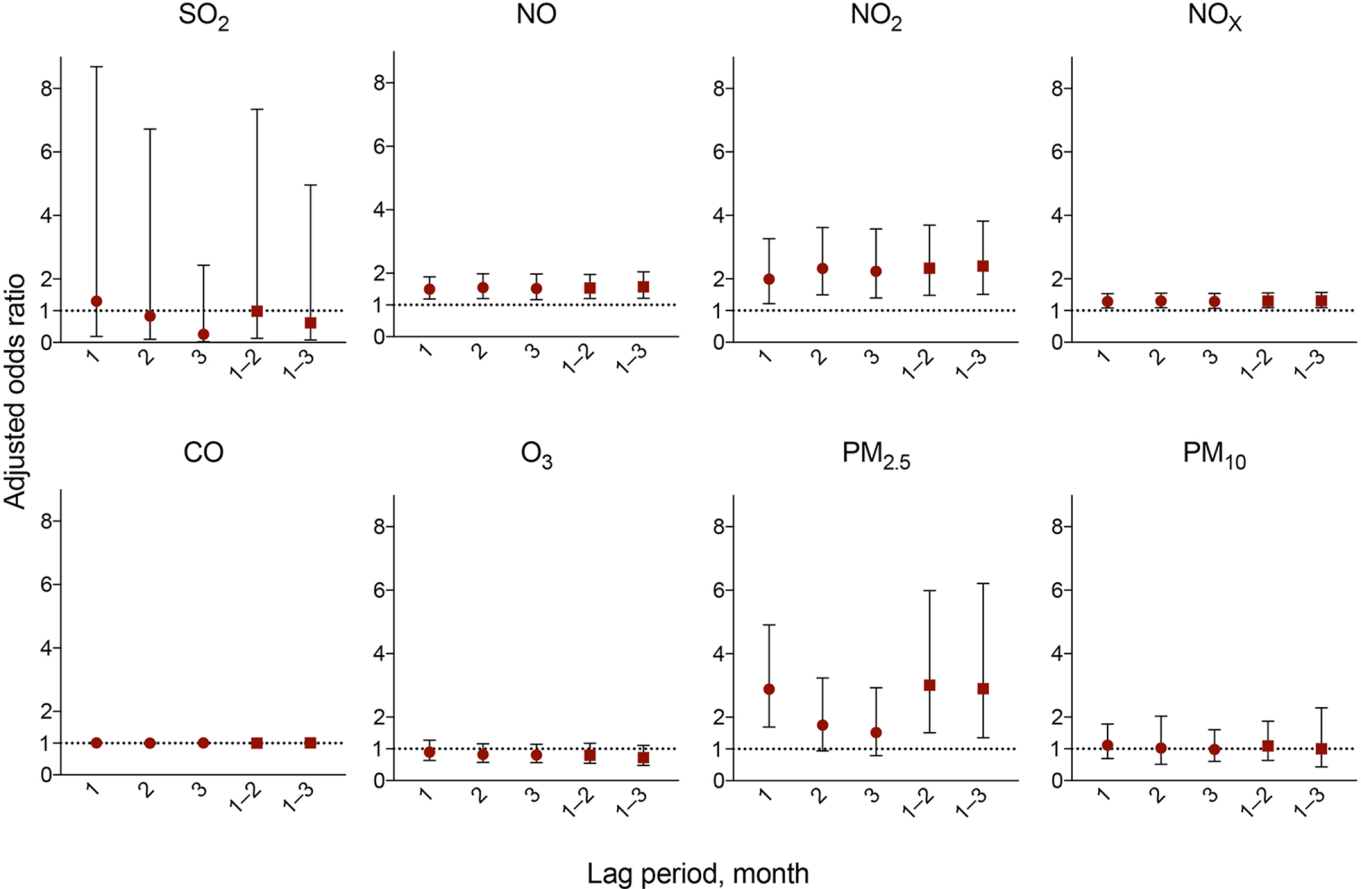
Adjusted ORs and 95% CIs for the incidence of AE-IIPs with different monthly lag periods. Adjusted odds ratios (ORs) and 95% confidence intervals (CIs) showing the increased risk of acute exacerbation of idiopathic interstitial pneumonias (AE-IIPs) associated with a 10-unit increase in the levels of sulfur dioxide (SO_2_), nitric oxide (NO), nitrogen dioxide (NO_2_), nitrogen oxides (NO_X_), carbon monoxide (CO), ozone (O_3_), particulate matter < 2.5 µm (PM_2.5_) and particulate matter < 10 µm (PM_10_) with different monthly lag periods. The adjusted ORs were estimated using multilevel mixed-effects logistic regression models, adjusting for temperature, humidity, age, sex, smoking status (pack-years) and percentage of the predicted value of forced vital capacity and diffusing capacity of the lung for carbon monoxide, and neighbourhood-level factors

The adjusted OR for AE-IPF associated with a 10 unit increase in exposure to NO was 1.46 (95% CI 1.11–1.93; p = 0.008) and PM_2.5_ was 2.56 (95% CI 1.27–5.15; p = 0.009) (Table 4). Figure 3 shows the results with different monthly lag periods for AE-IPF. Significant positive associations were observed between the monthly mean exposure to NO (lag 1, lag 2, lag 1–2, and lag 1–3), NO_2_ (lag 2 and lag 1–2), PM_2.5_ (lag 1 and lag 1–2), and AE-IPF (Fig. 3). The adjusted OR for AE-IIPs incidence associated with a 10 unit increase in exposure to NO was 1.50 (95% CI 1.19–1.88; p = 0.001), NO_2_ was 1.99 (95% CI 1.22–3.27; p = 0.006), NO_X_ was 1.29 (95% CI 1.08–1.53; p = 0.004), and PM_2.5_ was 2.88 (95% CI 1.69–4.91; p ≤ 0.001) (Table 5). Figure 4 shows the results with different monthly lag periods for AE-IIPs. Significant positive associations were observed between the monthly mean exposure to NO (lag 1, lag 2, lag 3, lag 1–2, and lag 1–3), NO_2_ (lag 1, lag 2, lag 3, lag 1–2, and lag 1–3), NO_X_ (lag 1, lag 2, lag 3, lag 1–2, and lag 1–3), PM_2.5_ (lag 1, lag 1–2 and lag 1–3), and AE-IIPs. (Fig. 4). Complete details of the results are available in the Additional file 1: Result.

**Table 4:** Association between exposure to air pollutants and the incidence of AE-IPF

Air pollutants	Increase	Adjusted OR	95% CI	p-value
SO_2_	10 ppb	0.35	0.03–3.88	0.39
NO	10 ppb	1.46	1.11–1.93	**0.008**
NO_2_	10 ppb	1.71	0.89–3.25	0.105
NO_X_	10 ppb	1.24	0.99–1.53	0.052
CO	10 ppb	1.01	0.99–1.02	0.52
O_3_	10 ppb	0.99	0.60–1.64	0.983
PM_2.5_	10 µg/m^3^	2.56	1.27–5.15	**0.009**
PM_10_	10 µg/m^3^	1.04	0.55–1.99	0.90

Results are presented as adjusted ORs and 95% CIs; the model was adjusted for temperature, humidity, age, sex, smoking status (pack-years), percentage of predicted value of FVC and DL_CO_ and neighbourhood-level factors. The adjusted ORs are presented per 10-unit increase in levels of SO_2_ (ppb), NO (ppb), NO_2_ (ppb), NO_X_ (ppb), CO (ppb), O_3_ (ppb), PM_2.5_ (µg/m^3^) and PM_10_ (µg/m^3^). p-values statistically significant are presented in bold

*AE-IPF* acute exacerbation of idiopathic pulmonary fibrosis, *OR* odds ratio, *CI* confidence interval, *FVC* forced vital capacity, *DL*_*CO*_ diffusing capacity of the lung for carbon monoxide, *SO*_2_ sulfur dioxide, *NO* nitric oxide, *NO*_2_ nitrogen dioxide, *NO*_X_ nitrogen oxides, *CO* carbon monoxide, *O*_3_ ozone, *PM*_2.5_ particulate matter < 2.5 µm, *PM*_10_ particulate matter < 10 µm

**Table 5:** Association between exposure to air pollutants and the incidence of AE-IIPs

Air pollutant	Increase	Adjusted OR	95% CI	p-value
SO_2_	10 ppb	1.30	0.19–8.69	0.79
NO	10 ppb	1.50	1.19–1.88	**0.001**
NO_2_	10 ppb	1.99	1.22–3.27	**0.006**
NO_X_	10 ppb	1.29	1.08–1.53	**0.004**
CO	10 ppb	1.01	0.99–1.03	0.16
O_3_	10 ppb	0.90	0.64–1.27	0.55
PM_2.5_	10 µg/m^3^	2.88	1.69–4.91	** < 0.001**
PM_10_	10 µg/m^3^	1.11	0.70–1.78	0.65

Results are presented as adjusted ORs and 95% CIs; the model was adjusted for temperature, humidity, age, sex, smoking status (pack-years), percentage of predicted value of FVC and DL_CO_ and neighbourhood-level factors. The adjusted ORs are presented per 10-unit increase in levels of SO_2_ (ppb), NO (ppb), NO_2_ (ppb), NO_X_ (ppb), CO (ppb), O_3_ (ppb), PM_2.5_ (µg/m^3^), and PM_10_ (µg/m^3^). p-values statistically significant are presented in bold

*AE-IPF* acute exacerbation of idiopathic pulmonary fibrosis, *OR* odds ratio, *CI* confidence interval, *FVC* forced vital capacity, *DL*_*CO*_ diffusing capacity of the lung for carbon monoxide, *SO*_2_ sulfur dioxide, *NO* nitric oxide, *NO*_2_ nitrogen dioxide, *NO*_X_ nitrogen oxides, *CO* carbon monoxide, *O*_3_ ozone, *PM*_2.5_ particulate matter < 2.5 µm, *PM*_10_ particulate matter < 10 µm

## Discussion

Based on the nationwide surgically diagnosed IPF registry, the present study demonstrated that there was a positive relationship between short-term monthly exposure to PM_2.5_ and the incidence of AE-IPF. An increase in 10 µg/m^3^ of PM_2.5_ amplified the risk of AE-IPF by approximately 2.5-fold.

In the previous two studies (South Korea and France), a significant positive association was observed between the increased levels of O_3_ and NO_2_ during 0–42 days prior to the diagnosis of AE-IPF [4, 14]. Moreover, these studies suggested potential (but not statistically significant) risks of elevated levels of PM_2.5_ (France) or PM_10_ (South Korea) for AE-IPF [4, 14]. Our study demonstrated that the increased mean level of PM_2.5_ 30 days before AE diagnosis has significant positive association with the increased risk of AE-IPF after adjusting for temperature, humidity, age, sex, smoking status, and percentage of the predicted value of FVC, DL_CO_, and neighborhood-level factors. On the other hand, no significant relationship was observed between AE-IPF incidence and exposure to NO_2_ and O_3_. The reason of the difference between our study and the previous studies on NO_2_ and O_3_ is unclear; however, the difference in the definition of IPF diagnosis, ethnicity, environment of studied areas and statistical methodology might explain that there was no relationship between these irritant pollutants and the incidence of AE-IPF in our study. In this study, the proportion of UIP pattern in HRCT was 15% of the IPF patients, which was less than that of the previous study [8]. Future studies are required to resolve these different findings.

Several in-vivo and in-vitro findings support the biological plausibility of the correlation between the elevated PM_2.5_ level and the incidence of AE-IPF [28]. PM_2.5_ tends to be deposited in the lower airways [28] inducing subsequent inflammation that could exacerbate asthma and COPD [29]. Furthermore, particulate matter causes mitochondrial damage in macrophages and produces reactive oxygen species, which can damage cellular proteins, lipids, membranes, and DNA [30–32]. Therefore, it was speculated that exposure to airborne PM_2.5_ triggers an inflammatory reaction and induces tissue damage in the lungs; thus, contributing significantly to AE-IPF.

Previous studies applied the exposure time window as “the 0–42 days” prior to the diagnosis of AE [4, 14] because the clinical course of AE-IPF should be within 1 month prior to AE diagnosis in the definition [9, 10]; however, the specific risk-exposure time window prior to the diagnosis of AE-IPF remains unclear. Significant positive associations between the exposure to increased PM_2.5_ levels and the AE-IPF diagnosis were observed during lag 1 and lag 1–2 months in our additional analysis. An increased mean level of PM_2.5_ during 15–21 days (OR 3.65; 95% CI 1.95–6.83; p ≤ 0.001) showed the strongest impact on AE-IPF (Additional file 1: Fig. S7) in our weekly analysis. These results suggest that “the 0–42 days” prior to AE-IPF diagnosis is useful in assessing the risk for AE-IPF regarding exposure to ambient PM_2.5_.

The health benefits of pollution reduction strategies have been documented in the Asia–Pacific Region, including Japan [33]. The Japanese government has passed a legislation to limit emissions in 2001, with a subsequent decrease in the mean levels of PM_2.5_ from 38 to 26 µg/m^3^ (2009) [33]. In our study, the mean ambient PM_2.5_ level in patients with IPF was 15.4 (Table 4). Our findings showed that elevated levels of PM_2.5_, albeit lower than the PM_2.5_ level of 26 µg/m^3^—the mean levels of PM_2.5_ established by the Japanese government in 2009—may be a risk factor for AE-IPF. Additionally, the mean exposure level of patients with IPF to PM_2.5_ was higher than the annual level recommended by the World Health Organisation (not exceeding an annual level of 10 µg/m^3^ for PM_2.5_) [34]. Accordingly, our findings suggest that the current ambient PM_2.5_ level remains a possible risk factor of AE-IPF in Japan.

We demonstrated a novel correlation between short-term monthly exposure to NO and AE-IPF. There was no report that exposure to ambient NO is a risk factor for respiratory diseases, including AE-IPF, presumably because few countries measure ambient NO in the atmosphere. In addition, significant negative association between AE-IPF and exposure to SO_2_ during lag 3 was observed. The negative correlation between exposure to SO_2_ and AE-IPF has been unclear. Further research is needed to determine the clinical significance of these results.

This study establishes the significant positive correlations between the AE-IIPs and short-term monthly exposure levels of NO, NO_2_, NO_X_, and PM_2.5_. This is the first study to show the correlation between AE-IIPs and short-term exposure to ambient air pollutants. Further research is necessary to clarify the relationship between exposure to air pollutants, including fine particulate matter and AE-IIPs.

The strengths of the present study are as follows. First, accurate IPF diagnoses were obtained in our study. All patients with IIPs participated in this study were diagnosed using SLB samples and MDD using a new video-conferencing system were performed [20]. Although SLB is not necessary in the diagnosis of IPF for all patients [1, 35], the most reliable method of diagnosing IPF is obtained from information on SLB and following MDD with ILD experts [36, 37]; this is because of an unignorable rate of commingling fibrosing lung diseases, such as fibrotic hypersensitivity pneumonitis [38]. Second, we adjusted the models for age, sex, and risk factors reported in previous studies (e.g., smoking status and percentage of the predicted value of FVC) [26] and time-varying variables (e.g., temperature and humidity).

Several limitations of this study should be acknowledged. First, this study was a retrospective study. Second, the sample size was relatively small and consisted only of Japanese patients; however, it comprised only patients with surgically diagnosed IPF, the most robust method for IPF diagnosis. Third, socioeconomic information that should be used for adjustment were not available in the database. Fourth, the addresses of patients were not available in the database; however, the hospital addresses were used as an alternative, because the addresses of patients are approximately 10 min away by ambulance from the nearby hospitals in Japan (Additional file 1: Table S7). In addition, all distances between the registered hospitals and monitoring stations were within 10 km (Additional file 1: Figs. S3 and S4–6). Fifth, we were unable to distinguish between the levels of air pollutant at home and in the workplace.

## Conclusions

The results of the case–control study suggest that short-term monthly exposure to PM_2.5_ may be a contributing risk factor to AE-IPF. We identified that the exposure periods of ambient PM_2.5_ recorded between 1 to 2-month prior to AE diagnosis were positively associated with AE-IPF in the additional analysis. Consistent with previous reports, we confirmed that “the 0–42 days” period preceding diagnosis may be useful in evaluating the relationship between the mean level of airborne PM_2.5_ and incidence of AE-IPF. Although the recent reduction in the levels of air pollutants has conferred health benefits, further efforts are required to decrease exposure to PM_2.5_ and reduce the risk of AE-IPF in Japan. We also identified the positive association between short-term exposure to NO and AE-IPF. Prospective cohort studies are expected to validate the relationship between exposure to ambient PM_2.5_, NO, and AE-IPF.

## Supplementary Information

**Additional file 1**. Supplementary Material.

## Data Availability

The datasets used for the current study are available from the corresponding author on reasonable request.

## Abbreviations

AE: Acute exacerbation
AE-IPF: Acute exacerbation of idiopathic pulmonary fibrosis
CI: Confidence interval
CO: Carbon monoxide
COPD: Chronic obstructive pulmonary disease
DL_CO_: Diffusing capacity of the lung for carbon monoxide
FVC: Forced vital capacity
HRCT: High-resolution computed tomography
IIPs: Idiopathic interstitial pneumonias
ILD: Interstitial lung disease
IPF: Idiopathic pulmonary fibrosis
KL-6: Krebs von den Lungen-6
MDD: Multidisciplinary discussion
NO: Nitric oxide
NO_X_: Nitrogen oxides
NO_2_: Nitrogen dioxide
OR: Odds ratio
O_3_: Ozone
SLB: Surgical lung biopsy
SO_2_: Sulfur dioxide
SP-D: Surfactant protein-D
SPM: Suspended particulate matter
PM_10_: Particulate matter < 10 µm
PM_2.5_: Particulate matter < 2.5 µm
