# Supplementary material for: Exposure to PM2.5 is a risk factor for acute exacerbation of surgically diagnosed idiopathic pulmonary fibrosis: a case–control study
Source: Respir Res. 2021 Mar 12;22:80. doi: 10.1186/s12931-021-01671-6 (PMC7955640; doi:10.1186/s12931-021-01671-6)
Supplement: Supplementary file 1 — Additional file 1. Supplementary Material. [file 12931_2021_1671_MOESM1_ESM.docx]

**Supplementary Material**

**Exposure to PM_2.5_ is a Risk Factor for Acute Exacerbation of Surgically Diagnosed Idiopathic Pulmonary Fibrosis: A Case–Control Study**

Masahiro Tahara M.D.^1^, Yoshihisa Fujino M.D., M.P.H., Ph.D.^2^, Kei Yamasaki M.D., Ph.D.^1^, Keishi Oda M.D., Ph.D.^1^, Takashi Kido M.D., Ph.D.^1,3^, Noriho Sakamoto M.D., Ph.D.^3^, Toshinori Kawanami M.D., Ph.D.^1^, Kensuke Kataoka M.D., Ph.D.^4^, Ryoko Egashira M.D., Ph.D.^5^, Mikiko Hashisako M.D., Ph.D.^6^, Yuzo Suzuki M.D., Ph.D.^7^, Tomoyuki Fujisawa M.D., Ph.D.^7^, Hiroshi Mukae M.D., Ph.D.^3^, Takafumi Suda M.D., Ph.D.^7^, Kazuhiro Yatera M.D., Ph.D.^1*^

^1^Department of Respiratory Medicine, University of Occupational and Environmental Health, Japan, Kitakyushu, Japan

^2^Department of Environmental Epidemiology, Institute of Industrial Ecological Sciences, University of Occupational and Environmental Health, Japan, Kitakyushu, Japan

^3^Department of Respiratory Medicine, Nagasaki University Graduate School of Biomedical Sciences, Nagasaki, Japan

^4^Department of Respiratory Medicine and Allergy, Tosei General Hospital, Seto, Japan

^5^Department of Radiology, Faculty of Medicine, Saga University, Saga, Japan

^6^Department of Anatomic Pathology, Graduate School of Medical Sciences, Kyushu University, Fukuoka, Japan

^7^Second Division, Department of Internal Medicine, Hamamatsu University School of Medicine, Hamamatsu, Japan

**^*^CORRESPONDING AUTHOR:** Kazuhiro Yatera, M.D., Ph.D.

Department of Respiratory Medicine, University of Occupational and Environmental Health, Japan, 1-1 Iseigaoka, Yahatanishi-ku, Kitakyushu-city, Fukuoka, 807-8555, Japan

E-mail: [yatera@med.uoeh-u.ac.jp](mailto:yatera@med.uoeh-u.ac.jp)

**TABLE OF CONTENTS**

**Supplementary Background**

**Supplementary Methods**

*Source database*

*Correlation between meteorological and air pollutant exposure levels*

*Definitions of control and case months*

*Weekly sensitivity analyses*

*Transport time for emergency patients*

**Supplementary Results**

*Correlation between meteorological and air pollutant exposure levels*

*Association between exposure to air pollutants and the incidence of AE in unadjusted analysis*

*Weekly sensitivity analyses*

*Transport time for emergency patients*

**Supplementary Discussion**

**Supplementary Figures**

Figure S1. Definition of the control and case months

Figure S2. Definition of the control and case periods with different monthly lag periods

Figure S3. Distances between the hospitals and monitoring stations

Figure S4. Detailed locations of the registered hospitals and the nearest air monitoring stations in Hokkaido, Kanto, and Kinki region

Figure S5. Detailed locations of the registered hospitals and the nearest air monitoring stations in Chubu region

Figure S6. Detailed locations of the registered hospitals and the nearest air monitoring stations in Chugoku, Kyushu, and Okinawa region

Figure S7. Weekly sensitivity analysis during 6 weeks before AE-IPF in the exposure levels of NO and PM_2.5_

Figure S8. Weekly sensitivity analysis during 6 weeks before AE-IIPs in the exposure levels of NO, NO_2_, NO_X_ and PM_2.5_

**Supplementary Tables**

Table S1. Characteristics of patients with IIPs at the time of SLB

Table S2. Distribution of meteorological and air pollutant exposure levels in patients with IIPs

Table S3. Pearson correlation coefficients between meteorological and air pollutant exposure levels of patients with IPF

Table S4. Pearson correlation coefficients between meteorological and air pollutant exposure levels of patients with IIPs

Table S5. Association between exposure to air pollutants and the incidence of AE-IPF in unadjusted analysis

Table S6. Association between exposure to air pollutants and the incidence of AE-IIPs in unadjusted analysis

Table S7. Average ambulance transport time for emergency patients managed in each fire department in Japan

**Supplementary References**

**Supplementary Background**

Recently, several studies have reported the existence of acute exacerbation (AE) of idiopathic interstitial pneumonias (AE-IIPs) other than idiopathic pulmonary fibrosis (IPF) [1-3]; however, the correlation between short-term exposure to air pollutants and the incidence of AE-IIPs remains unclear. Hence, in this study, we also determined whether the incidence of AE-IIPs correlated with an increased mean exposure level of eight air pollutants in the month including AE diagnosis or the 1-month before AE diagnosis. Moreover, we performed an additional analysis with different monthly lag periods to measure the exposure-risk time window for 3 months before the AE-IIPs diagnosis.

**Supplementary Methods**

*Source database*

Fujisawa et al. developed the nationwide cloud-based integrated database for IIPs [4]. They first administered a survey of the Japanese Respiratory Society to assess the number of patients diagnosed with IIPs at each institution from April 2009 to March 2014. In this survey, there were 13,598 patients with an institutional diagnosis of IIPs, of whom 1,311 had biopsy-proven IIPs in 159 institutions in Japan. Of these 159 institutions, 39 agreed to the study proposal and participated in the study. Finally, 465 patients with an institutional diagnosis of IIPs who had undergone high-resolution computed tomography and surgical lung biopsy (SLB) from April 2009 to March 2014 were included in the database. This retrospective study was approved by the institutional review boards of the Hamamatsu University School of Medicine, Hamamatsu, Japan (14-360) [4].

*Correlation between meteorological and air pollutant exposure levels*

Pearson correlation coefficients were used to measure the correlation between meteorological and air pollutant exposure levels of patients with IPF and IIPs.

*Definitions of control and case months*

In this case–control study, the control months were defined as all the single months during the date of SLB procedure to the date of death or censoring in patients without AE. In patients with AE, we served all the single months other than the case month as control months (Supplemental Figure S1). For each AE diagnosis, the case month was defined as 30 days before AE diagnosis.

Supplemental Figure S2 shows the definition of the control and case periods in the additional analysis with different monthly lag periods. For each lag case period, the control periods were used for all the single months or every two to three cumulative months in patients without AE. In patients with AE, the control periods were used for these periods other than the case lag periods (Supplemental Figure S2).

*Weekly sensitivity analyses*

For air pollutants that were significantly associated with AE in the main analysis, we conducted weekly sensitivity analyses to estimate the exposure time window of the highest risk 42 days before the diagnosis of AE. The mean weekly exposure levels were calculated during 6 weeks immediately after the date of diagnosis of AE-IPF and AE-IIPs (i.e., days 0–7, 8–14, 15–21, 22–28, 29–35, and 36–42).

*Transport time for emergency patients*

We obtained the average ambulance transport time from patients’ home to nearby hospitals for emergency patients by the population managed in each fire department in Japan from the database of the Japan Fire and Disaster Management Agency in 2015 (<https://www.fdma.go.jp>).

**Supplementary Results**

*Correlation between meteorological and air pollutant exposure levels*

Supplemental Table S3 shows correlation between meteorological and air pollutant exposure levels of patients with IPF. Nitric oxide (NO), nitrogen dioxide (NO_2_) and nitrogen oxides (NO_X_) were positively and strongly correlated. Ozone (O_3_) was negatively and weakly associated with NO, NO_2_ and NO_X_ and positively and weakly associated with particulate matter <2.5 µm (PM_2.5_). PM_2.5_ was positively and moderately associated with particulate matter <10 µm (PM_10_).

Supplemental Table S4 shows correlation between meteorological and air pollutant exposure levels of patients with IIPs. NO, NO_2_ and NO_X_ were positively and strongly correlated. O_3_ was negatively and weakly or moderately associated with NO, NO_2_ and NO_X_ and positively and weakly associated with PM_2.5_. PM_2.5_ was positively and moderately associated with PM_10_.

*Association between exposure to air pollutants and the incidence of AE in unadjusted analysis*

A 10 unit increase in the monthly mean exposure level of NO (OR = 1.57; 95% CI = 1.29–1.92; p = <0.001), NO_X_ (OR = 1.29; 95% CI = 1.07–1.54; p = 0.01), and PM_2.5_ (OR = 2.17; 95% CI = 1.16–4.07; p = 0.02) were significantly positively associated with an increased risk of AE-IPF in unadjusted analysis (Supplemental Table S5).

A 10 unit increase in the monthly mean exposure level of NO (OR = 1.66; 95% CI = 1.37–2.02; p = <0.001), NO_2_ (OR = 1.63; 95% CI = 1.08–2.54; p = 0.02), NO_X_ (OR = 1.34; 95% CI = 1.14–1.59; p = <0.001), and PM_2.5_ (OR = 1.97; 95% CI = 1.18–3.26; p = 0.01) were significantly positively associated with an increased risk of AE-IIPs in unadjusted analysis (Supplemental Table S6).

*Weekly sensitivity analyses*

The weekly sensitivity analysis results for AE-IPF in NO exposure levels and PM_2.5_ 6 weeks before AE diagnoses are demonstrated in Supplemental Figure S7. Adjusted ORs for NO mean exposure levels during 0–7, 8–14, 15–21, 22–28, 29–35, and 36–42, and adjusted ORs for PM_2.5_ during 0–7, 15–21, and 22–28 days before AE were significant. An increased NO mean level during 29–35 days (OR = 2.07; 95% CI = 1.05–4.11; p = 0.04) and PM_2.5_ during 15–21 days (OR = 3.65; 95% CI = 1.95–6.83; p = <0.001) showed the strongest impact on AE-IPF (Supplemental Figure S7).

The weekly sensitivity analysis results for AE-IIPs in the exposure levels of NO, NO_2_, NO_X_, and PM_2.5_ during 6 weeks before AE are demonstrated in Supplemental Figure S8. Adjusted ORs for the mean exposure levels of NO and NO_X_ during 0–7, 8–14, 15–21, 22–28, 29–35, and 36–42 days, and adjusted ORs for NO_2_ and PM_2.5_ during 0–7, 15–21, 22–28, 29–35, and 36–42 days before AE were significant. An increased NO mean level during 29–35 days (OR = 1.62; 95% CI = 1.27–2.06; p = <0.001), NO_2_ during 29–35 days (OR = 2.56; 95% CI = 1.66–3.95; p = <0.001), NO_X_ during 29–35 days (OR = 1.41; 95% CI = 1.18–1.68; p = <0.001), and PM_2.5_ during 0–7 days (OR = 3.89; 95% CI = 2.46–6.18; p = <0.001) showed the strongest impact on AE-IIPs (Supplemental Figure S8).

*Transport time for emergency patients*

Average ambulance transport time from patients’ home to the nearby hospitals for emergency patients in Japan was approximately 10 min (Supplemental Table S6).

**Figure S1. Definition of the control and case months**

Separated lines indicate a 1-month period. Each separated blue and red lines indicate control and case months, respectively. Patient_3 and patient_6 developed AE. The other patients never developed AE during the date of SLB procedure to the date of death or censoring. Patients who were alive without the incidence of AE on March 31, 2017 were censored. Patients lost to follow-up were censored at the date of last contact/follow-up.

AE: acute exacerbation; SLB: surgical lung biopsy

**Figure S2. Definition of the control and case periods with different monthly lag periods**

Separated lines indicate a 1-month or a 2-month period. Each separated blue and red lines indicate control and case periods, respectively. Patients who were alive without the incidence of AE on March 31, 2017 were censored. Patients lost to follow-up were censored at the date of last contact/follow-up.

AE: acute exacerbation; SLB: surgical lung biopsy

**Figure S3. Distances between the hospitals and monitoring stations**

**Figure S4. Detailed locations of the registered hospitals and the nearest air monitoring stations in Hokkaido, Kanto, and Kinki region**

The black boxes and red circles indicate the geographic location of the registered hospitals and the nearest air monitoring stations in the present study, respectively. All monitoring stations were located within a 10-km radius of the hospitals.

**Figure S5. Detailed locations of the registered hospitals and the nearest air monitoring stations in Chubu region**

The black boxes and red circles indicate the geographic location of the registered hospitals and the nearest air monitoring stations in the present study, respectively. All monitoring stations were located within a 10-km radius of the hospitals.

**Figure S6. Detailed locations of the registered hospitals and the nearest air monitoring stations in Chugoku, Kyushu, and Okinawa region**

 The black boxes and red circles indicate the geographic location of the registered hospitals and the nearest air monitoring stations in the present study, respectively. All monitoring stations were located within a 10-km radius of the hospitals.**Figure S7. Weekly sensitivity analysis during 6 weeks before AE-IPF in the exposure levels of NO and PM_2.5_**

Adjusted odds ratios and 95% confidence intervals showing the increased risk of an acute exacerbation of idiopathic pulmonary fibrosis associated with a 10-unit increase in nitric oxide mean levels and particulate matter <2.5 µm (PM_2.5_) during 6 weeks before the AE diagnosis

**Figure S8. Weekly sensitivity analysis during 6 weeks before AE-IIPs in the exposure levels of NO, NO_2_, NO_X_ and PM_2.5_**

Adjusted odds ratios and 95% confidence intervals showing the increased risk of an acute exacerbation of idiopathic interstitial pneumonias associated with a 10-unit increase in nitric oxide mean levels, nitrogen dioxide, nitrogen oxides, and particulate matter <2.5 µm (PM_2.5_) during 6 weeks before the AE diagnosis

**Table S1. Characteristics of patients with IIPs at the time of SLB**

|  |  | All | Acute exacerbation | No acute exacerbation |
| --- | --- | --- | --- | --- |
| Subjects | | 352 | 74 | 278 |
| Case or control months | | 14,280 | 74 | 14,206 |
| Age (years) | | 65 [59–70] | 66 [61–70] | 65 [59–70] |
| Male | | 230 (65%) | 50 (68%) | 180 (65%) |
| Pack-years | | 20 [0–42] | 22 [0–48] | 20 [0–42] |
| Pulmonary function tests | |  |  |  |
|  | FVC (% predicted) | 81.8 [70.7–94.2] | 79.8 [69.3–87.9] | 82.7 [70.7–96.6] |
|  | DL_CO_ (% predicted) | 69.5 [54.0–83.7] | 61.9 [46.1–76.9] | 72.1 [56.3–86.0] |
| HRCT pattern | |  |  |  |
|  | UIP | 28 (8%) | 9 (12%) | 19 (7%) |
|  | Possible UIP | 186 (53%) | 41 (56%) | 145 (52%) |
|  | Inconsistent with UIP | 138 (39%) | 24 (32%) | 114 (41%) |
| Antifibrotic therapy | |  |  |  |
|  | Pirfenidone | 90 (26%) | 30 (41%) | 60 (22%) |
|  | Nintedanib | 30 (9%) | 6 (8%) | 24 (9%) |
| Data presented as median [interquartile rage] or frequencies (%). | | | | |
| IIPs: idiopathic interstitial pneumonias; SLB: surgical lung biopsy; FVC: forced vital capacity; DL_CO_: diffusing capacity of the lung for carbon monoxide; ; HRCT: high-resolution computed tomography | | | | |
|  |  |  |  |  |
|  |  |  |  |  |

**Table S2. Distribution of meteorological and air pollutant exposure levels in patients with IIPs**

|  | Acute exacerbation | | | | No Acute exacerbation | | | |
| --- | --- | --- | --- | --- | --- | --- | --- | --- |
| Air pollutants | Mean | Min | Median | Max | Mean | Min | Median | Max |
| Temperature (°C) | 14.3 | -3.3 | 13.3 | 29.5 | 16.3 | -9.4 | 16.4 | 30.0 |
| Humidity (%) | 67.3 | 42.7 | 68.5 | 87.0 | 66.8 | 36.0 | 67.0 | 88.0 |
| SO_2_ (ppb) | 2.7 | 0.0 | 2.6 | 12.5 | 2.8 | 0.0 | 3.0 | 17.0 |
| NO (ppb) | 10.2 | 1.0 | 3.4 | 98.0 | 5.6 | 1.0 | 4.0 | 69.0 |
| NO_2_ (ppb) | 17.2 | 3.1 | 14.2 | 41.0 | 14.9 | 2.0 | 14.0 | 46.0 |
| NO_X_ (ppb) | 26.7 | 2.5 | 16.7 | 133.0 | 20.5 | 4.0 | 17.0 | 106.0 |
| CO (ppb) | 471.2 | 81.6 | 428.4 | 2101.0 | 411.4 | 100.0 | 400.0 | 1700.0 |
| O_3_ (ppb) | 27.7 | 6.2 | 27.2 | 52.1 | 29.2 | 5.0 | 28.0 | 59.0 |
| PM_2.5_ (µg/m^3^) | 16.6 | 4.8 | 16.0 | 42.6 | 15.3 | 1.6 | 14.8 | 43.6 |
| PM_10_ (µg/m^3^) | 20.3 | 7.7 | 19.3 | 37.7 | 20.7 | 6.0 | 20.0 | 51.0 |
| SO_2_: sulfur dioxide; NO: nitric oxide; NO_2_: nitrogen dioxide; NO_X_; nitrogen oxides; CO: carbon monoxide; O_3_: ozone; PM_2.5_: particulate matter <2.5 µm; PM_10_: particulate matter <10 µm | | | | | | | | |

**Table S3. Pearson correlation coefficients between meteorological and air pollutant exposure levels of patients with IPF**

| Air pollutants | IPF | | | | | | | | | |
| --- | --- | --- | --- | --- | --- | --- | --- | --- | --- | --- |
|  | Temperature | Humidity | SO_2_ | NO | NO_2_ | NO_X_ | CO | O_3_ | PM_2.5_ | PM_10_ |
| Temperature | – | – | – | – | – | – | – | – | – | – |
| Humidity | 0.63^**^ | – | – | – | – | – | – | – | – | – |
| SO_2_ | 0.09^**^ | −0.11^**^ | – | – | – | – | – | – | – | – |
| NO | −0.24^**^ | −0.17^**^ | 0.07^*^ | – | – | – | – | – | – | – |
| NO_2_ | −0.36^**^ | −0.42^**^ | 0.17^**^ | 0.74^**^ | – | – | – | – | – | – |
| NO_X_ | −0.32^**^ | −0.32^**^ | 0.13^**^ | 0.93^**^ | 0.93^**^ | – | – | – | – | – |
| CO | −0.30^**^ | −0.20^**^ | 0.05^**^ | 0.48^**^ | 0.46^**^ | 0.51^**^ | – | – | – | – |
| O_3_ | 0.17^**^ | 0.01 | 0.18 | −0.36^**^ | −0.30^**^ | −0.35^**^ | −0.14^**^ | – | – | – |
| PM_2.5_ | 0.11^**^ | 0.02 | 0.26^**^ | −0.01 | 0.07^**^ | 0.03^*^ | 0.25^**^ | 0.35^**^ | – | – |
| PM_10_ | 0.47^**^ | 0.23^**^ | 0.26^**^ | −0.02 | 0.05^**^ | 0.02 | 0.06^**^ | 0.29^**^ | 0.67^**^ | – |
| IPF: idiopathic pulmonary fibrosis; SO_2_: sulfur dioxide; NO: nitric oxide; NO_2_: nitrogen dioxide; NO_X_; nitrogen oxides; CO: carbon monoxide; O_3_: ozone; PM_2.5_: particulate matter <2.5 µm; PM_10_: particulate matter <10 µm | | | | | | | | | | |
| ^*^p < 0.05, ^**^p < 0.001 | | | | | | | | | | |

**Table S4. Pearson correlation coefficients between meteorological and air pollutant exposure levels of patients with IIPs**

| Air pollutants | IIPs | | | | | | | | | |
| --- | --- | --- | --- | --- | --- | --- | --- | --- | --- | --- |
|  | Temperature | Humidity | SO_2_ | NO | NO_2_ | NO_X_ | CO | O_3_ | PM_2.5_ | PM_10_ |
| Temperature | – | – | – | – | – | – | – | – | – | – |
| Humidity | 0.63^**^ | – | – | – | – | – | – | – | – | – |
| SO_2_ | 0.10^**^ | −0.08^**^ | – | – | – | – | – | – | – | – |
| NO | −0.26^**^ | −0.21^**^ | 0.02^*^ | – | – | – | – | – | – | – |
| NO_2_ | −0.35^**^ | −0.43^**^ | 0.10^**^ | 0.73^**^ | – | – | – | – | – | – |
| NO_X_ | −0.33^**^ | −0.35^**^ | 0.06^**^ | 0.92^**^ | 0.93^**^ | – | – | – | – | – |
| CO | −0.32^**^ | −0.21^**^ | 0.05^**^ | 0.45^**^ | 0.43^**^ | 0.47^**^ | – | – | – | – |
| O_3_ | 0.16^**^ | 0.004 | 0.05^**^ | −0.41^**^ | −0.30^**^ | −0.38^**^ | −0.13^**^ | – | – | – |
| PM_2.5_ | 0.09^**^ | 0.009 | 0.29^**^ | −0.04^**^ | 0.06^**^ | 0.01 | 0.26^**^ | 0.36^**^ | – | – |
| PM_10_ | 0.46^**^ | 0.22^**^ | 0.26^**^ | −0.05^**^ | 0.04^**^ | 0.002^**^ | 0.04^**^ | 0.28^**^ | 0.64^**^ | – |
| IIPs: idiopathic interstitial pneumonias; SO_2_: sulfur dioxide; NO: nitric oxide; NO_2_: nitrogen dioxide; NO_X_; nitrogen oxides; CO: carbon monoxide; O_3_: ozone; PM_2.5_: particulate matter <2.5 µm; PM_10_: particulate matter <10 µm | | | | | | | | | | |
| ^*^p < 0.05, ^**^p < 0.001 | | | | | | | | | | |

**Table S5. Association between exposure to air pollutants and the incidence of AE-IPF in unadjusted analysis**

| Air pollutants | Increase | OR | 95% CI | p-value |
| --- | --- | --- | --- | --- |
| SO_2_ | 10 ppb | 0.59 | 0.09–3.99 | 0.59 |
| NO | 10 ppb | 1.57 | 1.29–1.92 | <0.001 |
| NO_2_ | 10 ppb | 1.57 | 0.99–2.45 | 0.050 |
| NO_X_ | 10 ppb | 1.29 | 1.07–1.54 | 0.01 |
| CO | 10 ppb | 1.01 | 0.99–1.03 | 0.20 |
| O_3_ | 10 ppb | 0.87 | 0.63–1.21 | 0.42 |
| PM_2.5_ | 10 µg/m^3^ | 2.17 | 1.16–4.07 | 0.02 |
| PM_10_ | 10 µg/m^3^ | 0.81 | 0.49–1.33 | 0.40 |
| Results are presented as ORs and 95% CIs. The ORs are presented per 10-unit increase in levels of SO_2_ (ppb), NO (ppb), NO_2_ (ppb), NO_X_ (ppb), CO (ppb), O_3_ (ppb), PM_2.5_ (µg/m^3^) and PM_10_ (µg/m^3^).  AE-IPF: acute exacerbation of idiopathic pulmonary fibrosis; OR: odds ratio; CI: confidence interval; SO_2_: sulfur dioxide; NO: nitric oxide; NO_2_: nitrogen dioxide; NO_X_: nitrogen oxides; CO: carbon monoxide; O_3_: ozone; PM_2.5_: particulate matter <2.5 µm; PM_10_: particulate matter <10 µm | | | | |

**Table S6. Association between exposure to air pollutants and the incidence of AE-IIPs in unadjusted analysis**

| Air pollutants | Increase | OR | 95% CI | p-value |
| --- | --- | --- | --- | --- |
| SO_2_ | 10 ppb | 0.73 | 0.13–4.20 | 0.72 |
| NO | 10 ppb | 1.66 | 1.37–2.02 | <0.001 |
| NO_2_ | 10 ppb | 1.63 | 1.08–2.45 | 0.02 |
| NO_X_ | 10 ppb | 1.34 | 1.14–1.59 | <0.001 |
| CO | 10 ppb | 1.01 | 0.99–1.03 | 0.08 |
| O_3_ | 10 ppb | 0.75 | 1.56–0.99 | 0.04 |
| PM_2.5_ | 10 µg/m^3^ | 1.97 | 1.18–3.26 | 0.01 |
| PM_10_ | 10 µg/m^3^ | 0.79 | 0.52–1.21 | 0.28 |
| Results are presented as ORs and 95% CIs. The ORs are presented per 10-unit increase in levels of SO_2_ (ppb), NO (ppb), NO_2_ (ppb), NO_X_ (ppb), CO (ppb), O_3_ (ppb), PM_2.5_ (µg/m^3^) and PM_10_ (µg/m^3^).  AE-IPF: acute exacerbation of idiopathic interstitial pneumonias; OR: odds ratio; CI: confidence interval; SO_2_: sulfur dioxide; NO: nitric oxide; NO_2_: nitrogen dioxide; NO_X_: nitrogen oxides; CO: carbon monoxide; O_3_: ozone; PM_2.5_: particulate matter <2.5 µm; PM_10_: particulate matter <10 µm | | | | |

**Table S7. Average ambulance transport time for emergency patients managed in each fire department in Japan**

| Size of the fire departments | | Average transport time (min) |
| --- | --- | --- |
| All | | 11.5 |
|  | <50,000 | 17.4 |
|  | 50,000–100,000 | 14.6 |
|  | 100,000–300,000 | 12 |
|  | 300,000–700,000 | 10.5 |
|  | >700,000 | 9.8 |

**SUPPLEMENTARY REFERENCES**

1. Park IN, Kim DS, Shim TS, Lim CM, Lee SD, Koh Y, Kim WS, Kim WD, Jang SJ, Colby TV: Acute exacerbation of interstitial pneumonia other than idiopathic pulmonary fibrosis. *Chest* 2007;132:214-220.

2. Arai T, Kagawa T, Sasaki Y, Sugawara R, Sugimoto C, Tachibana K, Kitaichi M, Akira M, Hayashi S, Inoue Y: Heterogeneity of incidence and outcome of acute exacerbation in idiopathic interstitial pneumonia. *Respirology* 2016;21:1431-1437.

3. Miyamoto A, Sharma A, Nishino M, Mino-Kenudson M, Matsubara O, Mark EJ: Expanded acceptance of acute exacerbation of nonspecific interstitial pneumonia, including 7 additional cases with detailed clinical pathologic correlation. *Pathol Int* 2018;68:401-408.

4. Fujisawa T, Mori K, Mikamo M, Ohno T, Kataoka K, Sugimoto C, Kitamura H, Enomoto N, Egashira R, Sumikawa H, et al: Nationwide cloud-based integrated database of idiopathic interstitial pneumonias for multidisciplinary discussion. *Eur Respir J* 2019;53:1802243.

5. Collard HR, Moore BB, Flaherty KR, Brown KK, Kaner RJ, King TE, Jr., Lasky JA, Loyd JE, Noth I, Olman MA, et al: Acute exacerbations of idiopathic pulmonary fibrosis. *Am J Respir Crit Care Med* 2007;176:636-643.
